# Neodymium in the oceans: a global database, a regional comparison and implications for palaeoceanographic research

**DOI:** 10.1098/rsta.2015.0293

**Published:** 2016-11-28

**Authors:** Tina van de Flierdt, Alexander M. Griffiths, Myriam Lambelet, Susan H. Little, Torben Stichel, David J. Wilson

**Affiliations:** 1 Department of Earth Science and Engineering, Imperial College London, South Kensington Campus, London SW7 2AZ, UK; 2 Ocean and Earth Science, National Oceanography Centre Southampton, University of Southampton, Southampton SO14 3ZH, UK

**Keywords:** dissolved neodymium isotopes, dissolved neodymium concentrations, dissolved aluminium concentrations, seawater, geotraces, palaeoceanography

## Abstract

The neodymium (Nd) isotopic composition of seawater has been used extensively to reconstruct ocean circulation on a variety of time scales. However, dissolved neodymium concentrations and isotopes do not always behave conservatively, and quantitative deconvolution of this non-conservative component can be used to detect trace metal inputs and isotopic exchange at ocean–sediment interfaces. In order to facilitate such comparisons for historical datasets, we here provide an extended global database for Nd isotopes and concentrations in the context of hydrography and nutrients. Since 2010, combined datasets for a large range of trace elements and isotopes are collected on international GEOTRACES section cruises, alongside classical nutrient and hydrography measurements. Here, we take a first step towards exploiting these datasets by comparing high-resolution Nd sections for the western and eastern North Atlantic in the context of hydrography, nutrients and aluminium (Al) concentrations. Evaluating those data in tracer–tracer space reveals that North Atlantic seawater Nd isotopes and concentrations generally follow the patterns of advection, as do Al concentrations. Deviations from water mass mixing are observed locally, associated with the addition or removal of trace metals in benthic nepheloid layers, exchange with ocean margins (i.e. boundary exchange) and/or exchange with particulate phases (i.e. reversible scavenging). We emphasize that the complexity of some of the new datasets cautions against a quantitative interpretation of individual palaeo Nd isotope records, and indicates the importance of spatial reconstructions for a more balanced approach to deciphering past ocean changes.

This article is part of the themed issue ‘Biological and climatic impacts of ocean trace element chemistry’.

## 1. Introduction

Long-lived radiogenic isotope systems are characterized by slow decay of the parent isotope compared with the age of the Solar System. In the samarium–neodymium (Sm–Nd) system, radioactive ^147^Sm decays to the radiogenic daughter ^143^Nd with a half-life of 106 billion years. Ingrowth of ^143^Nd, together with differentiation of Sm and Nd during melting, has led to characteristic ^143^Nd/^144^Nd fingerprints in rocks, which vary as a function of age and lithology. Early applications of this decay system focused on geochronology, high-temperature geochemistry and cosmochemistry. The Nd isotopic composition is typically expressed in the epsilon notation (*ε*_Nd_), which denotes the deviation of a measured ^143^Nd/^144^Nd ratio from the bulk Earth/chondritic uniform reservoir (CHUR) ratio of 0.512638 in parts per 10 000 [1]. (We acknowledge that there is an ongoing discussion as to whether the bulk Earth actually has a chondritic composition for Sm/Nd (see Huang *et al.* [2] and references therein).)

In the late 1970s, the first direct measurements of Nd isotopes in seawater were accomplished [3,4]. Taken together with indirect seawater measurements, using ferromanganese crusts as archives [4–6], the picture soon emerged that seawater ^143^Nd/^144^Nd ratios show a clear provinciality between the different ocean basins [7]. The lowest ^143^Nd/^144^Nd ratios were observed in newly formed North Atlantic Deep Water (NADW, *ε*_Nd_ ∼ −13.5), the highest ratios occurred in North Pacific Deep Water (PDW, *ε*_Nd_ ∼ −4), and intermediate values were found in the deep Indian and Southern Oceans (*ε*_Nd_ ∼ −7 to −9). These observations pointed to a residence time of Nd in the deep ocean that is shorter than the deep water transit time between the North Atlantic and the North Pacific, giving rise to the promise of using Nd isotopes as a tracer for water mass sourcing and global ocean circulation ([8]; see also summaries by Goldstein & Hemming [9] and Frank [10]).

Reconstructions of authigenic (seawater-derived) Nd isotope records have been performed for a wide range of time scales and resolutions, from the Archaean to the present day. In particular, a growing number of Late Pleistocene records lend strong support to the notion that Nd isotopes may serve as a tracer for past water mass provenance and ocean circulation, particularly in the Atlantic Ocean [11–19]. However, quantitative use of the tracer is difficult at best, and simplifications have been necessary in order to derive (qualitative) interpretations of past changes. For example, it has often been assumed that the Nd isotopic composition of NADW is uniform and constant [8,9] and that mixing between water masses is largely conservative within an ocean basin. In contrast, recent studies have questioned the constancy of global endmembers through time [20–22] or pointed to non-conservative behaviour [23].

One important question is that of exactly *how* seawater acquires its Nd isotopic composition. This fundamental question is not yet fully resolved, although the general relationship with continental lithology is clear [24]. While early work suggested that the predominant inputs of Nd were via the dissolved riverine load and atmospheric dust [25,26], it has since emerged that the dissolved fluxes from rivers and dust alone are insufficient to close the mass balance of Nd in the ocean ([27,28], see also [29]). Interaction between sediments and seawater along continental margins (i.e. ‘boundary exchange’ [23,24]) is now thought to dominate the oceanic Nd budget [30–32]. In detail, both addition and removal of dissolved Nd can be observed during particulate–seawater exchange in marginal settings, involving processes such as adsorption/desorption, isotopic exchange or dissolution/precipitation [33]. One recent example from observational data is the dissolution of riverine particulate matter from the Amazon in the Atlantic Ocean [33,34]. Further suggestions for Nd additions to the ocean have included submarine groundwater discharge [35] and benthic fluxes from porewaters [36,37].

A further puzzle in the marine biogeochemical cycle of Nd and its isotopes is the partial decoupling between concentrations and isotopes, commonly referred to as the Nd paradox [9]. Away from ocean margins and areas of sluggish circulation, Nd isotopes in deep waters typically behave conservatively (i.e. they are only modified by mixing with other water masses). In contrast, Nd concentrations often show a (linear) increase with water depth, an observation that has been explained through the process of reversible scavenging [38,39]. However, recent results on dissolved rare-earth elements (REEs) from the CoFeMUG cruise in the South Atlantic [40], in conjunction with quantitative deconvolution of water masses, demonstrated that a large fraction (greater than 75%) of dissolved light REEs in deep waters are preformed. High bottom water concentrations in that region were explained by remineralization of particles, either *in situ* or along the flow path of Antarctic Bottom Water (AABW) [40].

In order to advance understanding of the interplay of biogeochemical and physical processes affecting dissolved Nd (and other REEs), and in order to provide a more grounded modern-day understanding for palaeo applications of Nd isotopes, Nd isotopes were designated as one of the key parameters in the international GEOTRACES programme (www.geotraces.org). GEOTRACES is a global study to understand the marine biogeochemical cycles of trace elements and their isotopes, and the sea-going phase of the programme was launched in 2010 [41]. Here, we present three different approaches to reflect on the status of pre-GEOTRACES and initial GEOTRACES work on dissolved Nd isotopes and concentrations in seawater, including their significance for palaeoceanographic reconstructions. We begin by presenting a new global database for seawater Nd isotopes and concentrations in the context of hydrography and macronutrients, via the GEOTRACES International Data Assembly Centre (and as electronic supplementary material). In the second part, we carry out a comparison of recently published Nd isotope and concentration data, hydrography, nutrients and Al concentration data from two of the first GEOTRACES transect cruises, in the northwest and northeast Atlantic Ocean. Finally, we reflect on the significance of our observations for the use of Nd isotopes as a palaeoceanographic tracer.

## 2. An updated global database for neodymium isotopes and concentrations

### (a) Rationale

Lacan *et al.* [42] published a global compilation of seawater Nd isotopes and concentrations, which included all published data up to 1 September 2011. It consisted of 880 Nd isotope data points, which were the primary selection criteria for the database (i.e. seawater stations where only REE or Nd concentrations were measured were not included) [42]. Here, we present a similar database, compiled independently and enlarged to contain all data published by 1 January 2016 (i.e. an additional approx. 1000 data points published between 2011 and 2015, which were collected outside the GEOTRACES programme and hence are not always contained in dedicated national and international data centres). Furthermore, and for the first time, to the best of our knowledge, we provide historical Nd isotope and concentration data alongside hydrography data extracted and calculated from the original publications (i.e. temperature, salinity, potential/neutral density and nutrient contents) to facilitate comparison of Nd isotopes and concentrations with basic hydrography.

The rationale for compiling and presenting this database follows from the GEOTRACES-driven shift in the way chemical oceanographic data are collected and archived. International GEOTRACES cruises are only approved as such if they facilitate the collection of a range of key trace element and isotope data (TEIs) [41]. Alongside these key TEIs, classical hydrography and nutrient measurements are routinely carried out. All data are subsequently archived and made publicly available through the GEOTRACES International Data Assembly Centre, hosted at the British Oceanographic Data Centre (www.bodc.ac.uk/geotraces/data). However, such a streamlined documenting and archiving process is a relatively new development for TEIs. Our compilation effort aims to close the gap between historical datasets and new GEOTRACES datasets, and thereby benefit the global community of chemical oceanographers, palaeoceanographers and modellers. A similar database for the full suite of dissolved REEs has been compiled by X. Zheng, Y. Plancherel and P. Scott (http://www.bodc.ac.uk/geotraces/data/historical/). While clearly beneficial towards understanding global Nd cycling, a full evaluation of combined seawater REE and Nd isotope data is beyond the scope of this study.

### (b) Observations

The new database is available under the following link: http://www.bodc.ac.uk/geotraces/data/historical/. We encourage the community to make us aware of any potential mistakes or oversights in the database.

**Figure 1.**
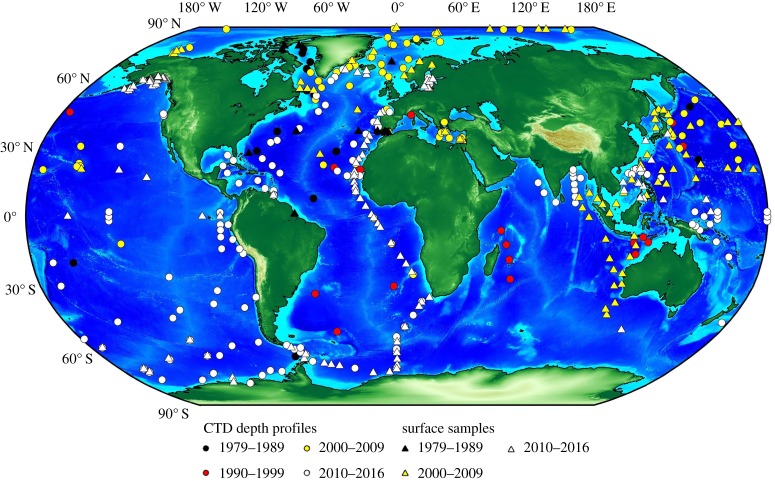
Map of global locations analysed for seawater Nd isotopes and concentrations (CTD = conductivity, temperature and depth). Circles correspond to depth profiles (i.e. three or more samples at one station) and triangles show surface samples only. Colour coding denotes the time period of data publication (see legend). Database compiled from [3,4,27,36,43–96].

As of January 2016, a total of approximately 2200 data points have been published for the Nd isotopic composition of seawater, which is more than double the amount of data available in 2011. In detail, figure 1 (and the online database) reveals that efforts in the 1980s and 1990s each contributed approximately 150 published data points, accomplished by a small number of laboratories. This number almost quadrupled in the 2000s (approx. 570 new data points) and more than doubled again in the last 5 years (approx. 1350 new data points). More seawater measurements have been carried out in the past 5 years than in the preceding 30 years. This major increase in data coverage since the 2000s can be partially attributed to the advance of multicollector inductively coupled plasma mass spectrometry (MC–ICP–MS), facilitating more rapid data generation, coupled to an increase in sea-going efforts and higher sampling resolution at each station. Together with the rising number of laboratories involved in the analysis of seawater Nd isotopic compositions and concentrations, this progress reflects the momentum generated by the launch of the GEOTRACES programme in 2006 [43,97]. Given that more than 50 GEOTRACES cruises (section cruises, process studies and international polar year cruises) have already taken place, with only partial Nd data from a handful of these cruises published so far, another pronounced increase in data coverage over the next 5–10 years is to be expected.

The database contains both filtered and unfiltered seawater samples. For operational reasons, many of the older data were generated from unfiltered seawater, whereas most of the newer data are being obtained from filtered seawater (typically 0.2–0.45 µm pore size filters). Given that particulate Nd only constitutes approximately 5% of the total seawater Nd budget [28,98,99], we would expect open ocean seawater to yield the same Nd isotopic composition in filtered and unfiltered samples [43]. However, this assumption may not be valid in areas with pronounced benthic nepheloid layers (BNLs), turbulent flow, high continental inputs and/or drastic differences between particulate and dissolved Nd isotopic compositions.

**Figure 2.**
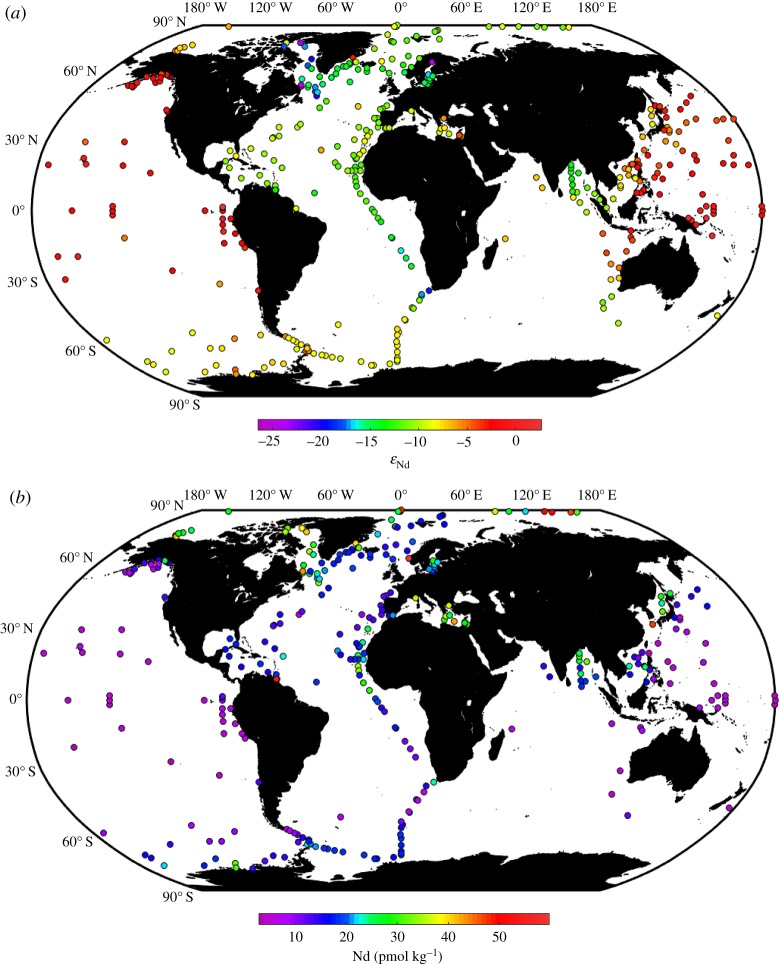
Map of surface (or shallow subsurface) seawater (*a*) Nd isotopic compositions and (*b*) Nd concentrations. Locations shown are restricted to those that yielded data within the top 100 m of the water column. Where measurements were made at multiple depths in one location, only the shallowest data are shown. In order to visualize the main features of the Nd distribution in the open ocean, stations with Nd concentrations greater than 60 pmol kg^−1^ are not shown. For references, see figure 1 and database.

Surface ocean Nd concentrations range from approximately 3 to approximately 60 pmol kg^−1^ (omitting three data points) and Nd isotopic compositions cover a range of 30 epsilon units from *ε*_Nd_ ∼ −27 to approximately +3 (excluding measurements directly at river mouths or in fjords). The lowest (i.e. least radiogenic) *ε*_Nd_ values are observed in the area of Baffin Bay (Labrador Sea, northwest Atlantic) and the highest (i.e. most radiogenic) *ε*_Nd_ values are found in the Pacific Ocean. When considering Nd concentrations, higher values ([Nd] > 20 pmol kg^−1^) are observed in surface waters proximal to continental input sources (e.g. Baffin Bay, Bay of Bengal, Mediterranean Sea). Surface waters in many areas of the Pacific Ocean show lower Nd concentrations ([Nd] < 10 pmol kg^−1^; figure 2), reflecting a lack of significant surface inputs to remote parts of this large ocean basin, as well as the efficient removal by particle scavenging in some marginal areas [44].

**Figure 3.**
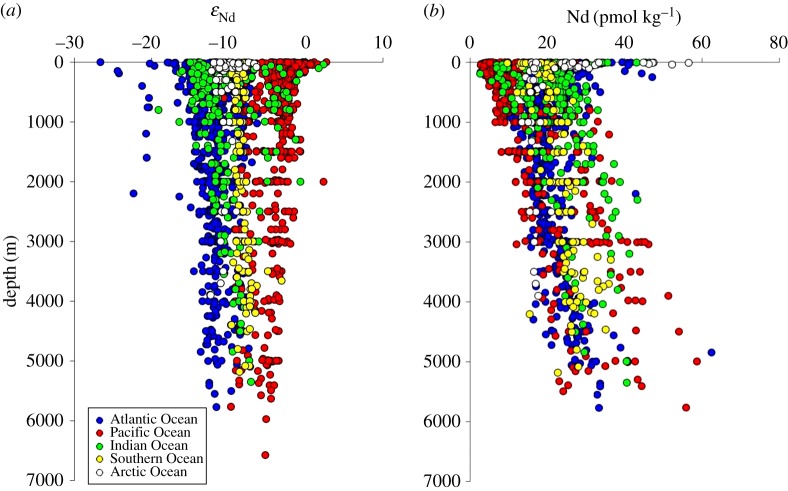
Global depth profiles for seawater (*a*) Nd isotopic compositions and (*b*) Nd concentrations. Colour coding corresponds to different geographical regions. For simplicity, the Arctic Ocean was defined as the area north of 78°N in the Atlantic realm, and north of 71°N in the Pacific realm. The Southern Ocean was defined as latitudes south of 60°S. Data from the marginal seas (i.e. Caribbean, Mediterranean, Baltic and South China seas) are not shown. For better visualization, six data points below 7000 m from the northwest Pacific Ocean [54] are not shown. For references, see figure 1 and global database.

**Figure 4.**
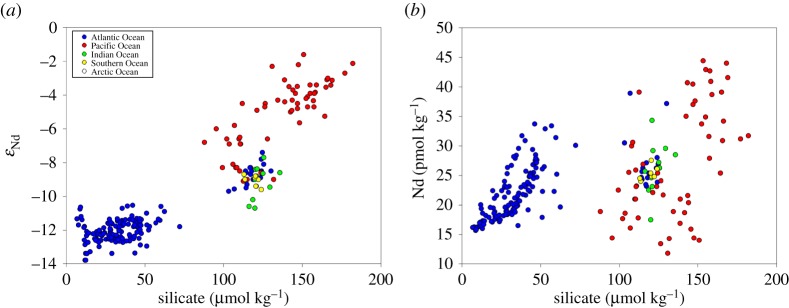
Published samples for which dissolved Si concentrations were available alongside (*a*) Nd isotopic compositions and (*b*) Nd concentrations from water depths below 2000 m. Note that high Nd concentrations are a persistent feature of bottom water layers around the world, and are not observed at all locations in the Pacific Ocean. For references, see figure 1 and global database.

Neodymium concentration profiles increase with water depth in most regions of the global ocean (figure 3*b*), with the exception of marginal seas and areas of strong convection and/or modified particle–seawater dynamics, which instead show invariant vertical concentration profiles (e.g. Arctic Ocean, Nordic Seas, Labrador Sea, Atlantic subpolar gyre, Caribbean, Mediterranean). The often-quoted statement that deep water Nd is generally lower in the North Atlantic and higher in the Pacific and correlates with silicate (see [9] and references therein) does seem to exclude data from the South Atlantic, Southern Ocean and Indian Ocean (figure 4). Zheng *et al*. [40] concluded that linear correlations of dissolved REEs and Si in Atlantic deep waters are due to predominantly conservative behaviour of both elements rather than similar chemical behaviour. This point is further supported by the data compiled in figure 4*b*. In fact, Atlantic deep waters describe different slopes in Si versus Nd space, with increasing Si and Nd concentrations with water depth. However, data from the Southern Ocean, Indian Ocean and Pacific Ocean do not continue the same trend and are rather scattered, particularly in areas where biogeochemical processes dominate over physical water mass advection (e.g. [44]; see also [39] and [100]).

## 3. Western and eastern North Atlantic GEOTRACES sections GA02 and GA03: neodymium, hydrography and nutrients

### (a) Study area

**Figure 5.**
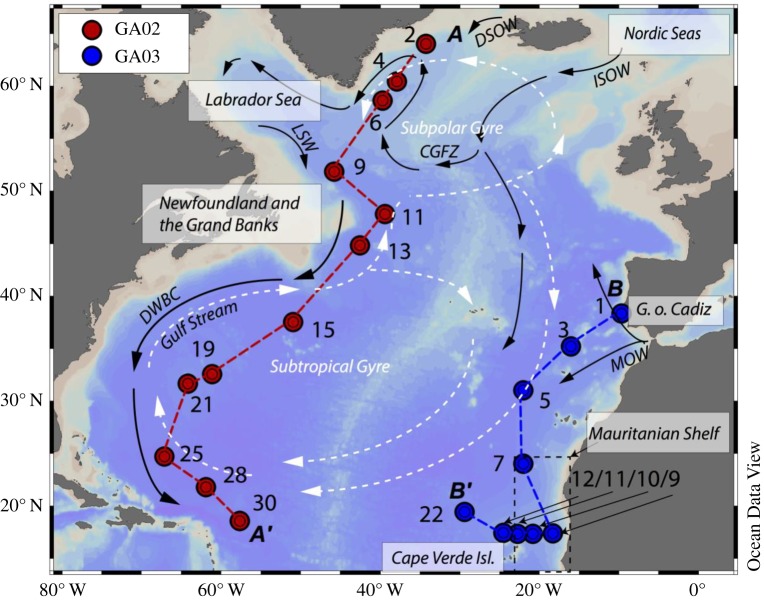
Stations analysed for Nd isotopic compositions and concentrations from Dutch GEOTRACES section GA02 (red) and US GEOTRACES section GA03 (blue), as well as geographical and hydrographic features in the North Atlantic. The main pathways of surface (white dashed arrows) and deep (black solid arrows) water masses are shown schematically. CGFZ, Charlie–Gibbs fracture zone; DSOW, Denmark Strait Overflow Water; DWBC, Deep Western Boundary Current; ISOW , Iceland–Scotland Overflow Water; LSW, upper and classical Labrador Sea Water; MOW, Mediterranean Outflow Water. Dashed lines labelled A to A′ and B to B′ illustrate the section lines of GA02 and GA03, respectively, shown in figures 6 and 9. Map visualized using the software Ocean Data View [101].

To take a closer look at the impact of vertical and lateral processes on the distribution of seawater Nd concentrations and isotopic compositions, we compare the more detailed results of two of the first full GEOTRACES section cruises, which sailed in the North Atlantic between 2010 and 2012 (figure 5). The meridional Dutch GEOTRACES section GA02 covers the full length of the Atlantic Ocean from east of Greenland to the southern Argentinian shelf. Sixty stations were sampled on four cruises between 2010 and 2012, and seawater Nd isotopes and concentrations were determined and published from 12 of the northernmost stations by Lambelet *et al*. [45] (figure 5). The US GEOTRACES section GA03 is located in the subtropical eastern North Atlantic and sailed in two legs in 2010 and 2011. The first leg sampled along a quasi-meridional section extending from Portugal to the coast of Mauritania and a short zonal section to the Cape Verde Islands. The second leg extended sampling from Woods Hole to the Cape Verde Islands in a zonal section across the subtropical North Atlantic. In total, 36 stations were sampled, and seawater from nine stations from the first leg in the eastern North Atlantic has been analysed for Nd isotopes and concentrations and published by Stichel *et al*. [46] (figure 5).

### (b) Hydrography

**Figure 6.**
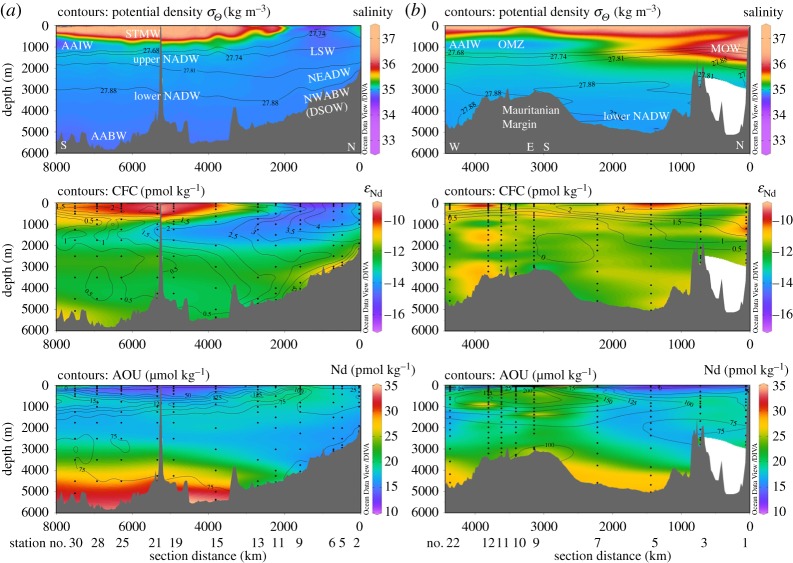
Hydrographic sections of (*a*) GA02 and (*b*) GA03 along A–A′ and B–B′, respectively (see figure 5 for section lines). Upper panel: salinity (coloured) with potential density anomaly relative to the surface isopycnals defining the main water masses; STMW, Subtropical Mode Water; (U)LSW = (upper) Labrador Sea Water; AAIW, Antarctic Intermediate Water; MOW , Mediterranean Outflow Water; NEADW, Northeast Atlantic Deep Water; NADW, North Atlantic Deep Water; NWABW, Northwest Atlantic Bottom Water; DSOW, Denmark Strait Overflow Water; and AABW, Antarctic Bottom Water. Middle panel: dissolved Nd isotopic compositions expressed as *ε*_Nd_ (coloured) with trichlorofluoromethane (CFC-11) isolines (CFC data from [45] and [102]). Bottom panel: dissolved Nd concentration (coloured) with apparent oxygen utilization (AOU) isolines. Numbers below the sections are station numbers. Data were visualized using the software Ocean Data View [101].

Detailed descriptions of section hydrographies are available from Jenkins *et al*. [102], Lambelet *et al*. [45] and references therein. Key features are highlighted in figures 5 and 6 and are summarized below.

Surface waters in both sections are influenced by the clockwise circulation in the subtropical gyre (approx. 10–40°N), and the anticlockwise flow of the subpolar gyre (north of approx. 50°N). Thermocline waters in the subtropical area are North Atlantic central waters (NACW; approx. 50–750 m depth), in their western and eastern varieties, including Subtropical Mode Water (STMW). Fresher and cooler Atlantic equatorial water dominates the thermocline of the Mauritanian upwelling part of the eastern section [102].

Upper Labrador Sea Water (ULSW) and classical Labrador Sea Water (LSW) are formed in the southern Labrador Sea (figure 5) and exported southwards at intermediate depth, where they constitute the upper part of NADW. Higher chlorofluorocarbon (CFC) concentrations along the upper NADW flow path in the Deep Western Boundary Current (DWBC) highlight the more recently ventilated parts of the water column in the west compared with the east (figure 6). A distinct feature at similar water depths and latitudes south of approximately 35°N in the eastern Atlantic Ocean is the Mediterranean Outflow Water (MOW), which is distinguished by its high salinity and high potential temperature (*S* = 36.4, *θ* = 12°C). Upon exit through the Straits of Gibraltar into the eastern Atlantic basin, MOW mixes with deeper Antarctic Intermediate Water (AAIW, approx. 800–1300 m), which, in turn, dominates the intermediate water column around the Mauritanian upwelling area ([102]; figure 6).

The deeper parts of NADW are comprised Iceland–Scotland Overflow Water (ISOW) and Denmark Strait Overflow Water (DSOW). Both these water masses are only present in rather dilute form in the North Atlantic, as they are modified from their source water compositions upon passing the sills separating the Nordic Seas from the open subpolar North Atlantic (figure 5), to form Northeast Atlantic Deep Water and Northwest Atlantic Bottom Water, respectively [103] (figure 6). While DSOW is detected in the northern stations along western section GA02, ISOW seems more significant for influencing deep water properties in the eastern basin [102]. In the subtropical deep Atlantic, both water masses are here combined and labelled as lower NADW (figure 6).

In the subtropical western and eastern North Atlantic, NADW flows southwards below AAIW and above AABW, two water masses formed in the Southern Ocean. Their northward export is more pronounced in the east, as traced by high concentrations of macronutrients, which stand out against nutrient-poor northern-sourced water masses (not shown).

In summary, the key differences between the western and eastern sections are (i) the strong flow associated with the DWBC in the western basin, and its associated strong export of LSW, (ii) the occurrence of MOW and an oxygen minimum zone (OMZ) off Mauritania in the eastern basin and (iii) the more pronounced influence of southern-sourced water in the east, and northern-sourced water in the west.

### (c) Neodymium concentrations, isotopic compositions and nutrients

Neodymium concentrations range between 15 and 25 pmol kg^−1^ in most of the intermediate to deep waters in both sections (figure 6). Lower values are observed in the surface and subsurface waters in the subtropics, whereas higher values are observed in surface waters close to the Mauritanian shelf and west of the Cape Verde Islands, as well as below approximately 4000 m water depth in both sections (figure 6). While maximum Nd concentrations in bottom waters in the eastern Atlantic are 28.5 pmol kg^−1^, slightly higher values of up to 33.4 pmol kg^−1^ are observed in bottom waters along the deeper southern part of the western transect. With the exception of the subpolar area (GA02) and the pronounced OMZ off Mauritania (GA03), Nd concentrations below 2000–2500 m increase almost linearly with water depth in both eastern and western basins.

Neodymium isotopic compositions in both sections are largely described by *ε*_Nd_ values between −11 and −13 (figure 6). Higher values are observed in subtropical (sub)surface waters, and lower values are displayed in a well-defined tongue of LSW expanding southwards in the western basin (*ε*_Nd_ = −13.7 ± 0.9) [45]. Overall, most stations along the eastern section reveal a rather homogeneous Nd isotopic composition with depth, whereas the profiles in the western section show pronounced patterns related to the prevailing water masses in the region of DWBC flow (i.e. outside the subpolar gyre). Notably, the typical composition of MOW (*ε*_Nd_ = −9.4 ± 0.6) [47] is only hinted at in the northernmost station in the Gulf of Cadiz (*ε*_Nd_ = −10.3 to −10.8 between 657 and 1482 m water depth; station 1 in figure 5). For reference, table 1 summarizes our best estimates for the source compositions of key intermediate and deep water masses in the North Atlantic.

**Table 1.** Source water masses with hydrographic characteristics.

abbreviations	full name	salinity	pot. temp. *Θ* (°C)	O_2_ (μmol kg^−1^)	silicate (μmol kg^−1^)	nitrate (μmol kg^−1^)	phosphate (μmol kg^−1^)	pot. dens. *σ*_θ_ (kg m^−3^)	*ε*_Nd_	Nd (pmol kg^−1^)	P*
AAIW	Antarctic Intermediate Water	34.139 ± 0.250	3.26 ± 0.52	257.4 ± 85.6	15.6 ± 5.5	27.5 ± 3.3	1.9 ± 0.1	27.17 ± 0.25	−8.0 ± 0.3	11.1	1.4
ULSW	Upper Labrador Sea Water	34.896 ± 0.015	3.84 ± 0.32	265.6 ± 5.6	9.3 ± 0.1	16.8 ± 0.1	1.1 ± 0.0	27.72 ± 0.02	−14.34 ± 0.13	18.5 ± 0.2	0.7
LSW	Labrador Sea Water	34.906 ± 0.023	3.45 ± 0.40	268 ± 3.1	10.6 ± 1.9	17.0 ± 0.3	1.1 ± 0.0	27.77 ± 0.06	−14.15 ± 0.07	18.1 ± 0.0	0.7
MOW	Mediterranean Outflow Water	38.462 ± 0.021	13.06 ± 0.06	189.9 ± 0.2	8.3 ± 0.6	8.6 ± 0.2	0.4 ± 0.0		−9.4 ± 0.6	23.3 ± 4.2	−0.5
ISOW	Iceland–Scotland Overflow Water	34.899 ± 0.007	−0.49 ± 0.36	317.0 ± 12.8	10.2 ± 1.2	12.9 ± 0.7	1.0 ± 0.1	28.03 ± 0.08	−8.2 ± 1.8	19.9 ± 5.3	0.9
DSOW	Denmark Strait Overflow Water	34.890 ± 0.028	−0.43 ± 0.59	315.5 ± 0.4	9.8 ± 4.4	13.9 ± 1.1	1.0 ± 0.1	28.02	−8.3 ± 0.2	15.9 ± 3.5	0.9
AABW	Antarctic Bottom Water	34.657 ± 0.016	−0.47 ± 0.45	234.9 ± 17.4	124.4 ± 3.6	33.3 ± 0.6	2.3 ± 0.0	27.85 ± 0.01	−9.1 ± 0.7	26.5 ± 2.4	1.7

The Nd isotopic composition and concentrations were chosen as the best guess for the endmember, so the closest to the source area of the water mass (i.e. by nature, most of these values are not sampled in sections GA02 and GA03). Where possible, the hydrographic data were taken for the same sample as the Nd data (e.g. AABW, AAIW, LSW and ULSW). Where the hydrographic data were not available for a corresponding Nd sample, they were taken from the following database: http://ocean.ices.dk/HydChem/HydChem.aspx?plot=yes and were chosen in order to match as closely as possible the salinity (and/or any other hydrographic data available) of the Nd sample.

The errors represent two sigma standard deviations.

AAIW: Neodymium and hydrographic data are from [86]: one sample from station 244 (200 m depth) and one from station 250 (500 m depth). The concentration was available for the sample from station 250 only. The samples were filtered, and collected between February and April 2008.

ULSW: Neodymium and hydrographic data are from the GA02 cruise [45]: two samples from station 9 (800 and 1000 m depth). Samples were filtered, and collected in May 2010.

LSW: Neodymium and hydrographic data are from the GA02 cruise [45]: two samples from station 9 (1250 and 1735 m depth). Samples were filtered, and collected in May 2010.

MOW: Neodymium data from [47]: two samples from station MED-15 (150/250 m and 400/500 m). Samples were filtered, and no collection data were available. Hydrographic data: ICES Data Centre, cruise 35JC, station 8, *n *= 5. Collected in October 1981.

ISOW: Neodymium data are from [49]: three samples from station SGN 23 (between 600 and 1000 m depth). Station 23 is situated above the sill of the Faroe Scotland gap and represents ‘proto ISOW’ (i.e. before the entrainment of LSW and modified North Atlantic water). The samples were not filtered, and concentrations reported are as if filtered (i.e. considering that 5% of Nd is present in particulate form). Samples collected in August 1999. Hydrographic data: ICES Data Centre, cruise 74SC, station 326, *n *= 3. Collected in September 1999.

DSOW: Neodymium data are from [50]: one sample from station SGN 55 (610 m depth). The Nd isotopic composition was measured on a filtered sample. However, because the concentration was not available for that sample, it was taken from an unfiltered sample collected at the same depth, and was calculated as if filtered (i.e. considering that 5% of Nd is present in particulate form). Sample collected in August 1999. Hydrographic data: ICES Data Centre, cruise 64BS, station 5422, *n *= 2. Collected in September 1999.

AABW: Neodymium and hydrographic data are from [86]: three samples from station 161 (between 2400 and 4400 m depth) and three samples from station 193 (between 2200 and 4800 m depth). The samples were filtered, and collected between February and April 2008. Note that the samples were collected in the Atlantic sector of the Southern Ocean, and they therefore represent Weddell Sea deep or bottom water. The Nd isotope characteristics are different for AABW from the Pacific or Australian/Indian sectors of the Southern Ocean.

**Figure 7.**
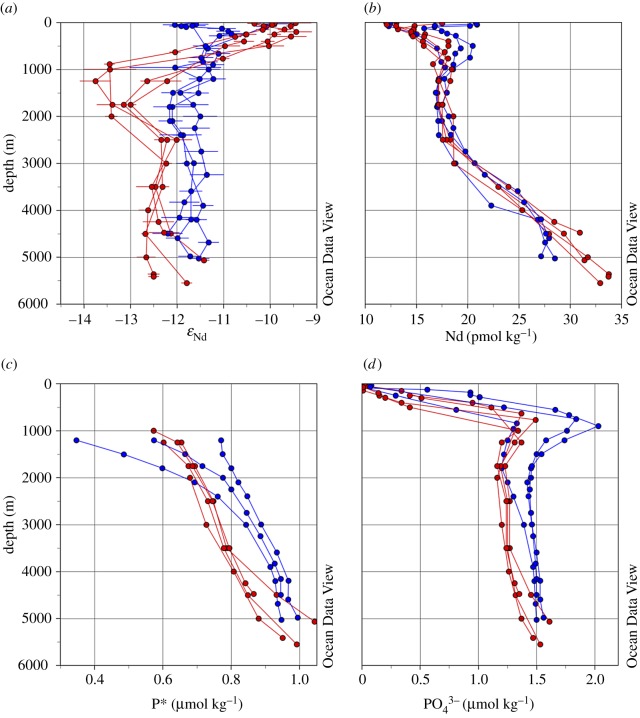
Selected depth profiles from sections GA02 (stations 15, 21, 25 and 30; red) and GA03 (stations 5, 7 and 22; blue) within the subtropical gyre, away from land masses: vertical distribution of seawater (*a*) Nd isotopic composition, (*b*) Nd concentration, (*c*) calculated phosphate star (P*) concentration below 1000 m and (*d*) phosphate concentration. Phosphate star is calculated after [104] using P* = PO_4_^3−^_observed_ + (O_2_/175) – 1.95. Oxygen and phosphate values for GA02 and GA03 were taken from the GEOTRACES IDP2014 [105]. Plots were generated using the software Ocean Data View [101].

Overall, seawater Nd concentrations and isotopic compositions are partially decoupled (i.e. the Nd paradox; figure 7). Neodymium concentrations correlate well with nutrients in the upper water column, owing to the adsorption of Nd onto (organic) particles and subsequent release upon remineralization [45,46]. The exceptions are areas of surface Nd input around Grand Banks (GA02; Labrador Current) and the Mauritanian shelf (GA03; dust input; figure 6). Minimum Nd concentrations are observed over the depth of the mixed layer, below which they start to increase alongside phosphate concentrations, consistent with the fact that approximately 50% of the phosphate in the upper water column is regenerated, and with the hypothesis that scavenged Nd is released when organic matter is remineralized. Notably, the phosphate maximum and the coincident Nd concentration maximum are both more strongly expressed in the eastern basin than in the western basin (figure 7). Below that level, Nd concentrations decrease slightly with depth, are fairly constant between 1000 and 2500 m depth, and then increase towards the bottom. These features are observed throughout the subtropics, with zonally remarkably similar Nd concentrations, in contrast to distinct phosphate concentrations in both sections (figure 7*b*; cf. figure 7*d*). The similarity in the Nd concentration profiles also differs from the rather distinct shapes of the Nd isotope profiles between the sections (figure 7*b*; cf. figure 7*a*), suggesting at least partially different controls on these properties.

If Nd isotopes can be used as a tracer for ocean circulation, then *ε*_Nd_ values should delineate the main deep water masses in a similar way to the conservative water mass tracer phosphate star (PO_4_^3–^*; also called ‘initial phosphate’, P*; where P* = PO_4_^3−^_observed_ + ( O_2_/175) – 1.95; [104]). Phosphate star expresses the preformed component of phosphate in the ocean, which behaves conservatively as long as the revised Redfield ratio of oxygen consumption to phosphate remineralization in the deep ocean is constant (175 : 1 [106,107]). In order to calculate the conservative tracer P*, nutrient data for the GA02 and GA03 cruises from the GEOTRACES intermediate data product (IDP2014) are used [105]. The east–west separation in phosphate concentrations is also visible for derived P* values (figure 7), indicating a greater contribution of southern-sourced water masses (higher P* values) in the middle and deep depths of the eastern basin compared with the western basin, consistent with hydrographic analyses [102].

**Figure 8.**
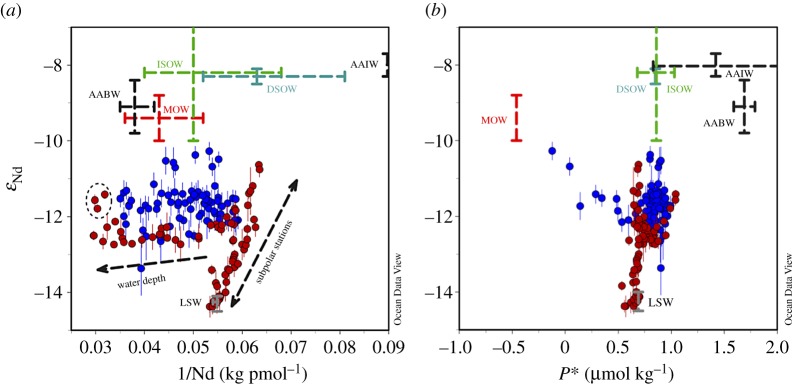
Neodymium isotopic composition as a function of (*a*) 1/Nd and (*b*) phosphate star (P*) for samples deeper than *σ*_0_ = 27.68 kg m^−3^ (i.e. ULSW and below) from sections GA02 (red symbols) and GA03 (blue symbols). The approximate endmember compositions of the discussed water masses are also illustrated (table 1). For abbreviations of water masses, see figure 6. A detailed description of the near-vertical and near-horizontal trends indicated in panel A is provided in the main text. The three circled points in that plot represent the samples most strongly influenced by AABW. Note that P* is not a good tracer to distinguish between the two different North Atlantic Deep Water source regions (i.e. LSW versus Nordic Seas overflows), whereas Nd isotopes do allow this distinction. In contrast, neodymium isotopes are not very different between overflow waters (i.e. DSOW and ISOW), MOW and Antarctic water masses, whereas the P* values of those water masses are distinct. Plots were generated using the software Ocean Data View [101].

Considering P*, *ε*_Nd_ and Nd concentration results from both sections for intermediate and deep water masses only, some interesting trends are observed (figure 8). First, a strong correlation is displayed for *ε*_Nd_ versus 1/Nd for all stations sampled in the subpolar gyre (GA02), and for intermediate depth stations in the subtropical gyre (GA02), describing a mixing line between LSW and DSOW (figure 8*a*). This strong correlation suggests conservative behaviour of both dissolved Nd isotopes and Nd concentrations in a significant fraction of the samples analysed from GA02. No such correlation can be observed for the GA03 data, mainly due to the absence of the distinct LSW and DSOW signature away from the DWBC. Second, both datasets describe rather horizontal trends in *ε*_Nd_ versus 1/Nd space due to an increase of Nd concentrations with depth, common to the entire subtropical Atlantic Ocean (figure 6). Deviations from this trend are due to the influence of Antarctic waters (both sections, but more pronounced in the east) and Mediterranean waters (eastern GA03 section only) with more radiogenic Nd isotopic compositions (figure 8*a*). The apparently contrasting behaviour of Nd isotopes between the two sections is further highlighted in a cross plot with phosphate star (figure 8*b*). The GA03 data are described by rather confined *ε*_Nd_ and P* values, with the exception of a trend towards the distinct MOW endmember at intermediate depths (i.e. negative P* and more radiogenic *ε*_Nd_ values; table 1), making it difficult to use either of the two proxies to depict individual water masses in the eastern basin. Even so, the generally higher P* values (figures 7*c* and 8*b*) do indicate a more pronounced admixture of southern-derived water masses (mainly AAIW [46,102]). In contrast, the GA02 data show well-defined trends between LSW, DSOW and AABW, consistent with stronger export of NADW and its precursor water masses along the DWBC and excellent water mass tracing by Nd isotopes [45].

In summary, intermediate and deep water Nd isotopic compositions along section GA02 in the western basin trace water masses (figure 6). The dominant feature along the western section is the very unradiogenic signature of LSW, which is strongly advected southwards in the Deep Western Boundary Current (figure 6). The relative homogeneity of seawater Nd isotopic compositions along section GA03 in the eastern basin is probably the result of less pronounced lateral admixture of water masses (i.e. more sluggish circulation, as indicated by low CFC-11 inventories [108]), as well as the absence of clear Nd isotope gradients between the water masses. The only water mass in the eastern Atlantic with the potential to create a tongue of distinct Nd isotopic compositions is MOW, which however does not show a strong fingerprint away from the Gulf of Cadiz (figure 6; see also §4). Similar Nd concentration profiles on both sides of the North Atlantic (figure 7*b*) argue against a stronger vertical supply of Nd in the east. The tracer P* is superior to Nd isotopes in depicting contributions of Southern Ocean waters, whereas Nd isotopes are the better tracer to distinguish between northwest and northeast Atlantic water sources.

## 4. Western and eastern North Atlantic GEOTRACES sections GA02 and GA03: insights from comparing Nd concentrations and isotopic compositions with Al concentrations

**Figure 9.**
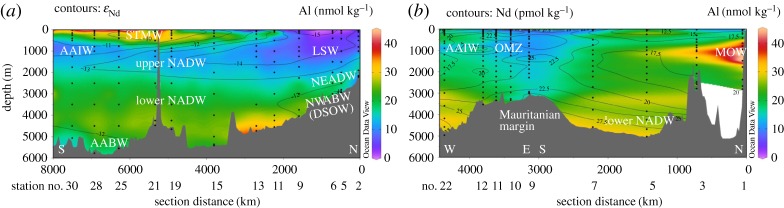
Aluminium concentrations along (*a*) section GA02 (line A–A′ in figure 5) contoured with Nd isotopic compositions and (*b*) section GA03 (line B–B′ in figure 5) contoured with Nd concentrations. For abbreviations of water masses, see figure 6. Numbers below the sections are station numbers. Data were visualized using the software Ocean Data View [101].

**Figure 10.**
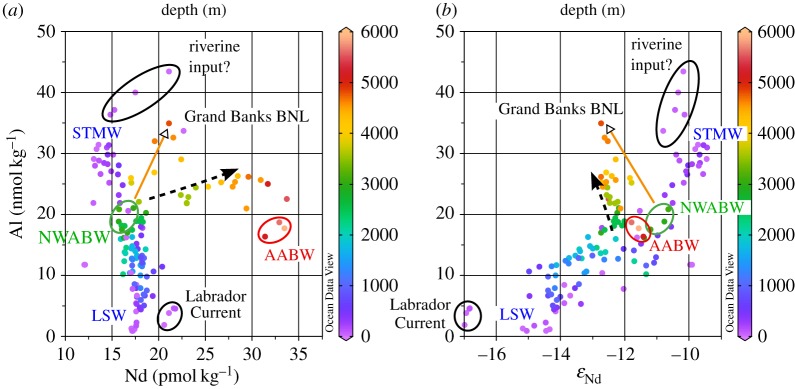
Aluminium concentrations versus (*a*) Nd concentrations and (*b*) Nd isotopic compositions for the western Atlantic section GA02. Colours represent water depth of sample collection. The main water masses are annotated and deviations from simple correlations are indicated by arrows or circles (see text for detailed description). Neodymium data are from [45] and Al data are from [109]. Plots were generated using the software Ocean Data View [101].

**Figure 11.**
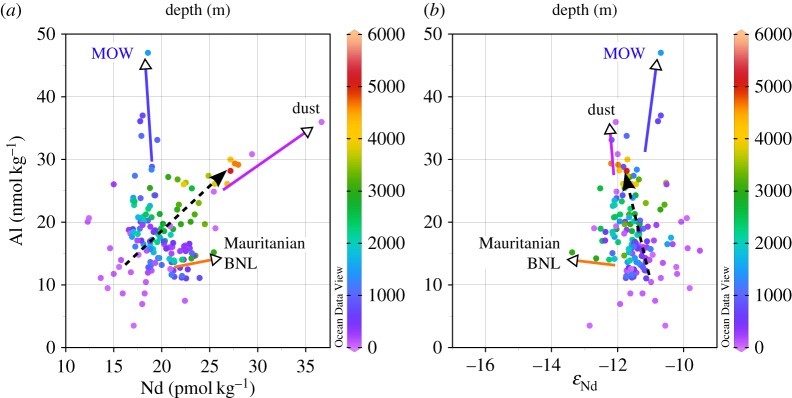
Aluminium concentrations versus (*a*) Nd concentrations and (*b*) Nd isotopic compositions for the eastern Atlantic section GA03. Colours represent water depth of sample collection. The only water mass that can be easily depicted in the area is MOW. Other arrows point towards the signature of dust influx and the Mauritanian benthic nepheloid layer (BNL). The dashed arrow indicates the trend of increasing Nd and Al concentrations with water depth. Neodymium data are from [46] and Al data are from [110]. Plots were generated using the software Ocean Data View [101].

The GEOTRACES programme is providing a wealth of data from equivalent samples for a large number of elements and isotope systems, beyond traditional measurements on hydrography and nutrients. Exploiting the potential for evaluation of trace metal cycling in ‘tracer–tracer’ space is, however, still in its infancy. In this section, we take a first step towards a tracer–tracer evaluation by carrying out a regional comparison of Nd isotopes and concentrations [45,46] with another lithogenic tracer, aluminium (Al). Aluminium data were collected on the same section cruises (GA02 and GA03), recently published by Middag *et al*. [109] and Measures *et al*. [110], and were extracted from the GEOTRACES IDP2014 [105]. There are many more Al concentration data for these sections than there are equivalent Nd concentration and isotope data, and only Al data for which equivalent Nd data points exist are included in the merged dataset. An offset of approximately 20% has been reported for the two Al datasets at the crossover station close to Bermuda (Bermuda Atlantic time series station, BATS), which remains to be explained, and prevents quantitative interpretations and illustration of Nd and Al data from the two sections (GA02 and GA03) on the same figures (figures 9–11).

As one of the first reactive trace elements to be reliably analysed in seawater, Al has a long history in oceanographic research [109–118]. However, despite many studies, both observational and modelling [119–121], the controls on oceanic Al distributions remain controversial. In the late 1990s to early 2000s, Al was thought of as the classic, short residence time, ‘scavenged’-type element, with high concentrations close to sources (predominantly atmospheric and benthic) and removal via particle scavenging [122]. Subsequent debate has focused on the relative roles of physical mixing versus internal cycling, and, in the latter case, passive scavenging versus active biological uptake. The arguments evolved around observations of Si–Al covariation (or its breakdown) and, in GEOTRACES data, the possibility of release (i.e. remineralization) of Al associated with high apparent oxygen utilization (AOU) in OMZs, as well as sedimentary (benthic) sources of Al. The possible importance of hydrothermal or hydrothermally related resuspension inputs of Al to the deep Atlantic has also been revisited [110].

### (a) Reversible scavenging versus water mass mixing

A major question for understanding the distribution of dissolved Al in the ocean is the extent to which internal cycling (i.e. scavenging and desorption or uptake and remineralization) is required to explain the observed distributions, or whether physical mixing of water masses is the main control [109,110]. Below the thermocline, both Nd and Al concentrations classically increase rather linearly with depth. Indeed, away from external sources (i.e. MOW), dust supply at the surface and the pronounced OMZ (i.e. Mauritanian margin), increases in Nd and Al concentrations with depth are observed on both sides of the Atlantic Ocean (figures 6 and 9). Reversible scavenging has been invoked as a mechanism for both elements, based on observational and modelling studies [38,39,119–121].

Models of reversible scavenging are based on the landmark study of Bacon & Anderson [123], which described the process for tracers with a radioactive decay source throughout the water column (e.g. ^230^Th). In the simpler case, for tracers without a radioactive decay source in the water column (e.g. Nd, Cu, Al), reversible scavenging models operate on the assumption that a single source of the tracer from the surface is balanced through removal by scavenging onto particles, according to an equilibrium scavenging coefficient, *K*,
4.1
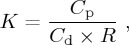

where *C*_d_ is the dissolved concentration of tracer per kg of seawater, *C*_p_ is the particulate-associated concentration of tracer per kg of seawater and *R* is the concentration of particulate material (and is dimensionless, being defined as the ratio of the mass of particles per cubic metre to the density of seawater). The equilibrium coefficient (*K*) is assumed to be constant with depth, and particles are assumed to have a constant sinking velocity. At steady state, the particulate-associated concentration of tracer (*C*_p_) must also remain constant with depth (i.e. what comes in at the surface goes out on particles). Rearranging equation (4.1),
4.2
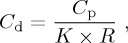

it becomes clear that, in order for *C*_p_ to remain constant as particles sink and dissolve or remineralize (i.e. while *R* decreases), the remaining particulate material must end up with a respectively *higher* concentration of adsorbed tracer (per kg of particle) and the dissolved concentration of tracer in seawater (*C*_d_) must increase. In addition, in this model, the slope of this dissolved concentration increase is dependent upon the specific particle dissolution profiles that are imposed. Note that if dust is the principal scavenger, and dust is assumed not to dissolve on sinking, a profile of constant C_d_ is also possible. This simple model does not account for the case of addition/formation of particles elsewhere in the water column, e.g., Fe or Mn oxides, which may also act as efficient scavengers.

### (b) Aluminium concentrations and neodymium isotopes in the western North Atlantic: physical circulation control?

Qualitatively, Al concentrations and Nd isotopic compositions in the western North Atlantic section share many features, which are evident in a section of Al concentrations with superimposed Nd isotope isolines (figure 9*a*). The distribution of Al in the western basin is strongly influenced by the distribution of the major water masses [109]. The radiogenic water mass in the subtropical thermocline (i.e. STMW) exhibits high Al concentrations due to a significant dust input to this region, whereas northern-sourced water masses of the subpolar gyre (ULSW and LSW) show much lower Al concentrations and an unradiogenic Nd isotope signature. These features of LSW reflect negligible Al input to the northwestern Atlantic surface waters, coupled with an Nd isotopic provenance signature acquired from the old continental crust surrounding this source region. In contrast, the source regions of the Southern Ocean-derived water masses (AAIW and AABW) are also regions of low Al input, but have much more radiogenic Nd isotope signatures, both of which are reflected in the resultant water mass compositions in section GA02. Finally, the striking distinction between upper NADW and lower NADW, documented for the first time in detail for Nd isotopes by Lambelet *et al*. [45], is equally well defined in Al concentrations, which are significantly elevated in lower NADW compared with upper NADW (figure 9*a*).

The elevated Al in lower NADW may in part be supplied directly from the overflow areas (e.g. DSOW) and increasing ‘ingrowth’ of Al concentrations from benthic sources along its flow path [109], but could also be linked to locally pronounced particle resuspension (and dissolution) associated with the BNL around Grand Banks (figure 9*a*). The peak in bottom water Al concentrations (at stations 11 and 13, approx. 45°N) is associated with a parallel shift towards the less radiogenic Nd isotope signature of lower NADW compared with its source components such as DSOW, suggesting active boundary exchange in this area.

These observations are also resolvable on cross plots of Al concentrations with Nd isotopes for the northwest Atlantic meridional section (figure 10*b*). Aluminium concentrations and Nd isotopic compositions in the upper approximately 2500 m of the water column show a strong positive correlation (purple to dark green colours in figure 10*b*). The most extreme compositions along this array are (i) northern-sourced ULSW/LSW, with low Al concentrations (less than 5 nmol kg^−1^ [109]) and unradiogenic Nd isotopic compositions (*ε*_Nd_ = − 14.3 ± 0.3 and −13.7 ± 0.9 [45]) and (ii) STMW, with high Al concentrations (greater than 25 nmol kg^−1^ [109]) and more radiogenic Nd isotopic compositions (*ε*_Nd_ = −9.6 ± 0.2 [45]). Notably, both endmembers are upper ocean water masses, with ULSW/LSW being formed by seasonal convection in the Labrador Sea before being exported at mid-depths as upper NADW.

Two locations in the surface ocean show deviations from the above-described trend (figure 10). The first region, comprising the most southerly stations 25, 28 and 30, has elevated Al concentrations in surface waters, associated with slightly less radiogenic Nd isotopic compositions and higher Nd concentrations than typically observed for STMW (circled black, figure 10). The principal source of both Al and Nd to the surface ocean in the subtropical Atlantic Ocean is dust, whereas Lambelet *et al*. [45] noted a drop in salinity in these particular samples and suggested the Caribbean rivers could provide an additional input source in this area. The second region showing deviation from the LSW–STMW correlation comprises the surface samples around Grand Banks (circled black, figure 10), indicating inflow of very unradiogenic Nd via the Labrador Current [45]. Neodymium concentrations are slightly elevated, but Al concentrations are very low (less than 5 nmol dm^−3^) in these waters (figure 10*a,b*), confirming that continental supply of Nd from old North American source terrains, via dissolved and/or particulate riverine material, does not seem to be a significant source for Al.

Additional groupings and trends between Al and Nd concentrations or isotopes can be observed in deeper waters (i.e. below 3000 m; light green to yellow-orange and red colours in figure 10). Northwest Atlantic Bottom Water (circled green, figure 10*b*), derived from its low Al and radiogenic Nd DSOW precursor, can be identified deep in the Irminger Basin (stations 2–6) and just to the north of Grand Banks (station 9) and lies on the LSW–STMW trend. However, its composition is modified on encountering the Grand Banks BNL, evolving towards somewhat less radiogenic *ε*_Nd_ values and highly enriched Al (and Nd) concentrations (orange arrows, figure 10). In the deep (depths below 3000 m) subtropical part of the section (stations 15–30), Nd and Al concentrations increase with water depth, whereas Nd isotopic compositions remain relatively unchanged. This trend could be interpreted either as upward diffusion of Nd and Al from a benthic source or as downward supply through reversible scavenging. The southernmost stations 25–30 encounter AABW, which is characterized by low Al concentrations, high Nd concentrations and a more radiogenic Nd isotopic composition (circled red, figure 10).

In summary, relatively simple mixing relationships can be distinguished between Al and Nd isotopes in the western North Atlantic (figures 9 and 10), suggesting that, away from source regions, Al behaves rather conservatively in the subsurface western North Atlantic. This observation does not rule out a minor role for internal processes, such as reversible scavenging or even limited scavenging loss of Al in deep waters, as proposed by Middag *et al*. [109].

### (c) Aluminium and neodymium concentrations and neodymium isotopic compositions in the eastern North Atlantic: disentangling physical mixing and internal cycling

In the eastern North Atlantic, the relationship of Nd isotopic compositions with water mass distribution is less well defined than in the western basin [46] (see §3). Aluminium concentrations in the eastern basin are strongly influenced by the presence of MOW, which is a very Al-enriched source [110]. Mediterranean Outflow Water is also identifiable by its more radiogenic Nd isotopic signature and can be clearly depicted on cross plots of Al concentrations with Nd isotopic compositions and Nd concentrations (figure 11), despite its Nd concentration being indistinct from surrounding water masses. It is interesting to note that the southward penetration of MOW is better resolved in the Al data (figure 9) than in Nd isotopic compositions (figure 6), whereas the opposite was observed for northwest Atlantic bottom water (NWABW) (i.e. DSOW). This observation may in part reflect the highly distinct Al signature of MOW (see also P*; figure 8*b*), but could also suggest that, under certain conditions, Al concentrations might even behave more conservatively than Nd isotopic compositions. Indeed, Measures *et al*. [110] showed convincingly that, for densities between 27.5 and 27.9 kg m^−3^, dissolved Al concentrations and salinity describe a mixing line between highly saline MOW and relatively fresh AAIW, indicating conservative behaviour of Al in this density layer.

However, there is also clear evidence that dissolved Al concentrations are influenced by internal cycling, in particular in the (sub)surface layer and near the continental margins. Both Al and Nd exhibit concentration minima associated with the chlorophyll maximum (in both the eastern and western Atlantic sections), interpreted to reflect scavenging on organic particles. Aluminium is also incorporated directly into diatom frustules [124–127], which would bolster this signature, although evidence for the significance of incorporation versus scavenging is inconclusive [120]. Below the chlorophyll maximum, Nd concentrations increase in step with AOU, indicating desorption associated with particle remineralization [46], a signature that is more pronounced in the stronger OMZ of the eastern basin compared with the western Atlantic [45] (figure 6). Paradoxically, a similar process was proposed for Al in the western Atlantic [109], but discounted in the eastern basin [110]. Whereas Nd concentrations are elevated in a broad plume within the eastern OMZ, Al concentrations do not display this pattern (figure 9), indicating that any source of Al from remineralization associated with OMZs must be minor and restricted to a second-order role in controlling the Al distribution. Instead, close to the Mauritanian upwelling region, Al concentrations are low throughout the water column, pointing towards persistent removal through scavenging.

One further interesting observation in the eastern section is the behaviour of Al in the Mauritanian BNL. As described in §4*b*, a significant benthic source of Al is associated with the permanent BNL at Grand Banks, concomitant with a minor source of Nd and a notable shift in Nd isotopic compositions (figure 10). A BNL was also identified at the Mauritanian margin at station 9, and is associated with an increase in Nd concentrations and a shift towards less radiogenic Nd isotopic compositions [46]. There is, however, no clearly resolvable impact on local dissolved Al concentrations (figure 11). This difference in the behaviour of Al in BNLs has been noted previously [109,110,116,117], and has been attributed to varying Si concentrations in ambient deep waters. High Si concentrations, for example associated with AABW, are believed to inhibit the re-dissolution of Al from suspended sediment [120,128], which could explain the lack of a dissolved Al signature in the Mauritanian BNL. This effect has also been invoked to explain why the strong Al source around Grand Banks (§4*b*) occurs at a water depth of approximately 5000 m (45°N), and slightly to the north of the densest nepheloid layer (depths below 5500 m, 40°N), because the latter location exhibits slightly elevated Si concentrations due to the greater presence of AABW [109]. More generally, the observation that the presence of a BNL is not always associated with an Al source calls into question the utility of deep- and bottom water Al concentrations as a tracer of boundary exchange processes for other elements. More work is needed to fully understand the control of BNLs on both elements, Al and Nd.

In summary, the Al distribution in the eastern section is more complex to interpret, in part because the seawater Nd isotope signal does not allow for simple water mass depiction, complicating a coupled evaluation of the two tracers. There remains ample evidence that Al may behave largely conservatively, particularly in the case of the invasion of MOW at thermocline depths [110]. However, scavenging of Al (and Nd) occurs in association with the subsurface peak in biological productivity, and the covarying (increasing) concentrations of Nd and Al in the deep eastern basin (figure 11*a*, dashed arrow) may yet hint at a role for reversible scavenging (or other internal cycling processes) in deep and bottom waters. Measures *et al*. [110] proposed a hydrothermal origin for the high Al concentrations in the deep eastern basin, but no such hydrothermal source can be invoked to explain high concentrations of Nd in the area [40,129,130].

## 5. Implications from GEOTRACES observations for palaeoceanographic applications

A better understanding of the processes influencing the cycling of Nd in the modern ocean offers the potential to improve the application of Nd isotopes for understanding past ocean changes. In this section, we discuss the implications from GEOTRACES observations for palaeoceanographic studies, focusing on the North Atlantic region and on the topics of water mass endmember compositions, boundary exchange and internal cycling.

### (a) North Atlantic water mass endmember compositions

Many palaeoceanographic studies assume a working model for the Atlantic Ocean that involves mixing between an unradiogenic NADW endmember with *ε*_Nd_ ∼ −13.5 and southern-sourced AABW and AAIW with more radiogenic values of *ε*_Nd_ ∼ −7 to −9 [8,9]. In this simplified view, northern values are set by weathering inputs from the old continental terrains surrounding the North Atlantic, and southern values are derived from admixture of more radiogenic Pacific waters into the Southern Ocean [8,9].

In detail, however, the northwest and northeast Atlantic source waters that contribute to NADW are distinct in their Nd isotopic composition [48]. Table 1 provides an update on the characteristics of the main intermediate to deep waters in the Atlantic Ocean. Labrador Sea Water is the most unradiogenic water mass in the North Atlantic (*ε*_Nd_(ULSW) = −14.34 ± 0.13 and *ε*_Nd_(LSW) = −14.15 ± 0.13), but its density is not high enough for it to sink beyond intermediate water depths (upper NADW). On the other hand, overflows from the Nordic Seas (*ε*_Nd_(ISOW) = −8.2 ± 1.8 and *ε*_Nd_(DSOW) = −8.3 ± 0.2; table 1) are sourced from more radiogenic surface waters (figure 2), and are furthermore influenced by exchange with young volcanic material in the area [49,50]. Upon export from the subpolar region, the overflows form the middle and lower layers of NADW, sometimes combined as lower NADW [45]. Data from GA02 have revealed that upper and lower NADW in the subtropical North Atlantic maintain an isotopic difference (upper NADW, *ε*_Nd_ = −13.2 ± 1.0; lower NADW, *ε*_Nd_ = −12.4 ± 0.4 [45]; figure 6).

**Figure 12.**
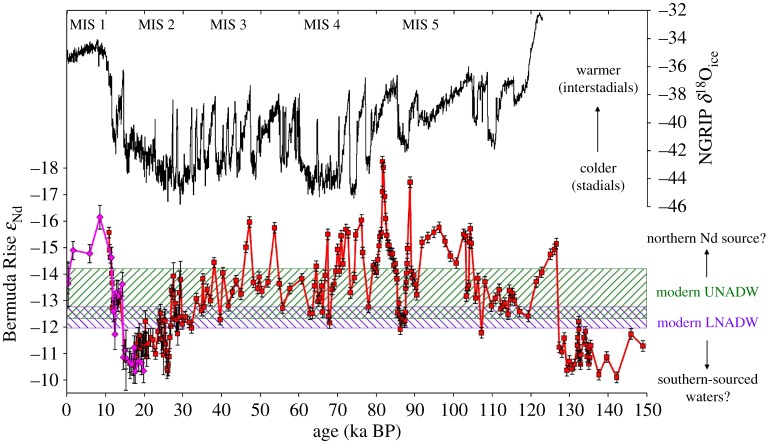
Time series of Nd isotope reconstructions through the last glacial cycle from Bermuda Rise cores OCE326-GGC6 [13] (pink) and ODP 1063 [15] (red) recovered from approximately 4500 m water depth. Also plotted (black) is the oxygen isotope record from Greenland ice core NGRIP [135], together with marine isotope stage (MIS) numbers. Modern-day Nd isotope endmembers are shown by hatched bars [45]. More radiogenic values during glacial maxima are widely linked to increased contributions from southern-sourced waters, whereas the origin of the highly unradiogenic values during warm periods is discussed in the text (see also [22]).

Even small differences in the Nd isotopic compositions of NADW source waters may become more critical going back in time. In the past oceans, there is significant uncertainty in where exactly deep waters formed in the North Atlantic, including a debate over the presence and activity of LSW and overflows from the Nordic Seas (i.e. ISOW, DSOW) during the last glacial period. It has been suggested that LSW formation was reduced during glacial periods and probably absent at the Last Glacial Maximum (LGM) [131], whereas past activity of the Nordic Seas overflows is controversial, with arguments for continued but episodic overflow activity during the glacial period [132,133] or a complete switch to open ocean convection south of Greenland [134]. Changes in the location of convection and/or mixing proportions of source waters during past climate events may have resulted in a different Nd isotopic composition for NADW or its glacial equivalent, Glacial North Atlantic Intermediate Water (GNAIW), and some existing records point in this direction. For example, Nd isotope values at approximately 4500 m depth on the Bermuda Rise (figure 12) were more extreme than any values in the modern deep Atlantic both during the Early Holocene (*ε*_Nd_ = −16.5) [13] and during interstadials of the last glacial period (*ε*_Nd_ = −15 to −18) [15]. The only feasible source regions for such negative values (i.e. old source rocks) are the continents and shelf areas around the Labrador Sea [24]. Consequently, Bohm *et al*. [15] interpreted such values to represent a larger proportion of LSW in exported NADW during warm times. However, this scenario is only feasible if LSW significantly deepened compared to today, requiring rearrangement of the density structure in the North Atlantic [22]. In contrast, during the coldest times of the LGM, it has been suggested that GNAIW may have had a more radiogenic Nd isotopic composition than modern NADW as a result of reduced LSW contributions [136].

Rather than viewing such complexity as a challenge for using Nd isotopes, it appears that a better spatial sampling of the glacial ocean could provide improved constraints on the changing locations of deep water convection and the activity of LSW and overflows from the Nordic Seas, particularly if partnered with other proxies such as oxygen isotopes, carbon isotopes, Pa/Th ratios or radiocarbon.

### (b) Boundary exchange

Boundary exchange describes particulate–seawater exchange occurring by a variety of processes, typically (but not exclusively) in marginal settings, and has been proposed as a major source of Nd to seawater [23,30,137]. If sediment–seawater interaction leads to the supply of Nd and/or isotopic exchange close to water mass source regions, then it can be considered as a mechanism for imprinting water masses with their Nd isotopic signatures [48], but it would not affect the ability of Nd isotopes to monitor water mass mixing further downstream, as indicated by modelling studies [31,100]. However, if benthic supply [36,37] and/or remineralization of particulate Nd along the flow path of a water mass are included [40,109], then those processes may provide a non-conservative influence that alters the Nd isotopic composition of bottom waters and hence the record obtained from sediment cores. Such processes would limit (but not remove) the potential of using Nd isotopes to reconstruct (past) ocean circulation.

The new GEOTRACES data discussed in §3 contribute to this debate by clarifying the extent and geographical locations of boundary exchange in the North Atlantic. In the modern ocean, there is evidence for active boundary exchange in the deep Irminger Sea near Greenland, and on the Grand Banks near Newfoundland, confirming previous suggestions that boundary exchange in these regions contributes to the generation of the NADW endmember composition [48]. For example, NWABW changes by 1.1 *ε*_Nd_ units over a distance of approximately 650 km in the Irminger Sea [45]. In contrast, there is a change of less than 1 *ε*_Nd_ unit over approximately 5500 km between stations 9 and 30 following the downstream flow of NADW in the western Atlantic basin (GA02 section), restricting boundary exchange to a relatively minor influence. However, it should be noted that AABW entrainment south of 45°N leads to a loss of contact of lower NADW with the seafloor [109]. Overall, water mass advection clearly dominates the Nd isotope signature in intermediate to deep waters in the western North Atlantic, and Nd isotope distributions conform well to those of conservative water mass tracers such as salinity, CFCs (figure 6) or neutral density [45]. Similarly, in the eastern Atlantic section (GA03), an imprint of boundary exchange on dissolved Nd isotopes was only implicated in two locations [46]: station 9 on the Mauritanian margin, where the BNL represents a net source of Nd, and station 12 near the Cape Verde Islands, where there is no accompanying change in Nd concentrations.

While previous studies have emphasized the role of continental shelves and ocean margins as major boundary sources, the examples above highlight the significance of localized benthic sources. In modelling studies, boundary exchange is typically parametrized by a function that is at its maximum in the surface layer and decreases exponentially with depth to zero below 3000 m [30,31,138]. With the exception of a slightly higher spatial variability in Nd isotopes in the (sub)surface layer of the global oceans (figure 3), no obvious depth dependence of boundary exchange has emerged as yet from GEOTRACES data. If such an observation proves to be robust across multiple settings, then this particular model assumption should probably be revised (see also §4). In addition, the mechanisms and processes involved in boundary exchange need further exploration, and particularly BNLs may act as an as yet understudied source (or sink) of trace metals in the ocean (see also [37]).

### (c) Internal cycling

Adsorption of Nd onto the surfaces of sinking particles and desorption upon decomposition may, in places, make dissolved Nd profiles in the upper ocean resemble those of biologically used elements (figure 7), albeit reflecting a rather different process [45,46]. Deeper in the water column, Nd concentrations at the depth levels of LSW (upper NADW), MOW and AAIW show remarkably conservative behaviour, an observation that has also been made for dissolved Al concentrations (see §4). In a recent study, Zheng *et al*. [40] used a multiparameter model to deconvolve the relative importance of water mass mixing and vertical cycling on seawater Nd concentrations along a section at 12°S in the Atlantic Ocean. They found that more than 75% of the REE concentrations (including Nd) are controlled by preformed concentrations, through physical transport and water mass mixing, and hence behave conservatively. However, away from water mass formation areas in the subpolar North Atlantic, Nd concentrations in excess of simple water mass mixing are observed in deeper waters and particularly in bottom waters (§§3 and 4), which may be tentatively attributed to reversible scavenging. Vertical transport by a reversible scavenging process may also play an important role in generating the homogeneous Nd isotopic distribution in the eastern Atlantic [46], where advection is more sluggish.

Excess Nd, observed in deep and bottom waters of the South Atlantic, has been attributed to either *in situ* remineralization or the accumulation of Nd along the flow path of southern-sourced bottom waters [40]. Based on the data from section GA02, where similar Nd concentrations in bottom waters are observed over approximately 4000 km of longitudinal transport, we would argue that along-flow accumulation is not widely occurring, and that *in situ* processes, such as the regeneration of Nd related to local calcite dissolution, are probably a more likely candidate. However, there is a gradient of increasing Nd concentrations along GA02 outside the subpolar gyre at the neutral density level of NWABW (i.e. lower NADW; approx. 3500–4500 m water depth) which does hint at the addition of Nd along the flow path of this particular water mass (see fig. 7 in [45]). Internal cycling, where it occurs, may potentially contribute to some downward smoothing of vertical Nd isotope structure, in comparison with a conservative tracer such as salinity, but from the GA02 and GA03 data, it does not appear to leave a clear Nd isotope fingerprint in this region. Future studies will need to tackle modern vertical Nd cycling in more detail before speculations on the nature of internal cycling in the past oceans can be made.

## 6. Concluding remarks

Our comparison of the recent GEOTRACES data published from the North Atlantic sections GA02 and GA03 [45,46] highlights similarities as well as differences in Nd cycling between the eastern and western Atlantic basins. Physical mixing seems to dominate the patterns observed in both Nd isotopes and concentrations in strongly advective areas, whereas deviations from mixing are observed in localized areas of non-conservative boundary exchange in both sections. Another feature common to both sections is the increase in Nd concentrations with water depth, generating rather similar concentration profiles for deep and bottom waters throughout the subtropical area. Along the western North Atlantic section, Al concentrations and Nd isotopes show strikingly similar behaviours, further supporting the notion that both tracers are excellent water mass indicators in this area. Interestingly, continental inputs from North America and Greenland, which dominate the fingerprint of LSW for Nd isotopes (and concentrations), are not significant sources of Al. Furthermore, in detail, Nd and Al concentration profiles reflect a variety of different processes superimposed on the first-order control of water mass mixing, including passive cycling of Nd with nutrients in the upper water column, benthic sources of both Nd and Al, and the potential for (reversible) scavenging to play a role in their distributions.

One clear implication of the modern GEOTRACES data is that a spatial context is important for palaeoceanographic studies, which is nicely illustrated in studies on sediment core material from the Holocene western Pacific [139] and the Holocene and glacial western Indian Ocean [140]. However, at present, the palaeo data generally do not have the spatial resolution (both in location and especially in water depth) of the new GEOTRACES data, and significantly more progress is warranted in that respect. Obtaining a depth perspective from the past oceans will require the integration of multiple archives, such as shallow-dwelling corals in the surface and subsurface oceans [141], deep-sea corals in intermediate depths [142,143], foraminifera [51,144,145] or sediment leachates [11,146] from mid-depth sites, and fish teeth [147,148] from abyssal settings. For this combined approach to be successful, datasets measured on different types of archives in different laboratories worldwide need to be reliable and comparable. That has been a challenging task, particularly in the case of sediment leachates, but considerable progress has been made [145,146,149–152].

More generally, the GEOTRACES data demonstrate that potential changes in endmember compositions, boundary exchange or internal cycling challenge quantitative interpretations of authigenic Nd isotopes as a circulation tracer. However, the qualitative information on water mass provenance and mixing gained from palaeo Nd isotope datasets can be significantly enhanced by: (i) studying analogue modern-day scenarios; (ii) taking multi-proxy approaches; (iii) increasing the regional and vertical coverage of records; and (iv) using modelling approaches to invert palaeo data.

## Supplementary Material

Global Database

## Data accessibility

The new global database on dissolved Nd isotopes, Nd concentrations and hydrography is available through the GEOTRACES International Data Assembly Centre, hosted at the British Oceanographic Data Centre (http://www.bodc.ac.uk/geotraces/data/historical/).
